# Supplementary material for: Development of intensiometric indicators for visualizing N-cadherin interaction across cells
Source: Commun Biol. 2022 Oct 7;5:1065. doi: 10.1038/s42003-022-04023-2 (PMC9546846; doi:10.1038/s42003-022-04023-2)
Supplement: Supplementary file 2 — Supplementary information [file 42003_2022_4023_MOESM2_ESM.pdf]

Supplementary information

Development of intensiometric indicators for visualizing N-cadherin interaction across cells

Takashi Kanadome[1,2], Kanehiro Hayashi[3], Yusuke Seto[4], Mototsugu Eiraku[4,5], Kazunori Nakajima[3], Takeharu Nagai[2] & Tomoki Matsuda[2*].

[1] Precursory Research for Embryonic Science and Technology (PRESTO), Japan Science and Technology Agency (JST), Kawaguchi, Saitama, 332-0012, Japan

[2] Department of Biomolecular Science and Engineering, SANKEN (The Institute of Scientific and Industrial Research), Osaka University, 8-1 Mihogaoka, Ibaraki, 567-0047, Japan

[3] Department of Anatomy, Keio University School of Medicine, Shinjuku-ku, Tokyo 160-8582, Japan

[4] Laboratory of Developmental Systems, Institute for Life and Medical Sciences, Kyoto University, Kyoto 606-8507, Japan

[5] Institute for the Advanced Study of Human Biology (WPI-ASHBi), Kyoto University, Kyoto 606-8507, Japan

*Corresponding author. Email: tmatsuda@sanken.osaka-u.ac.jp

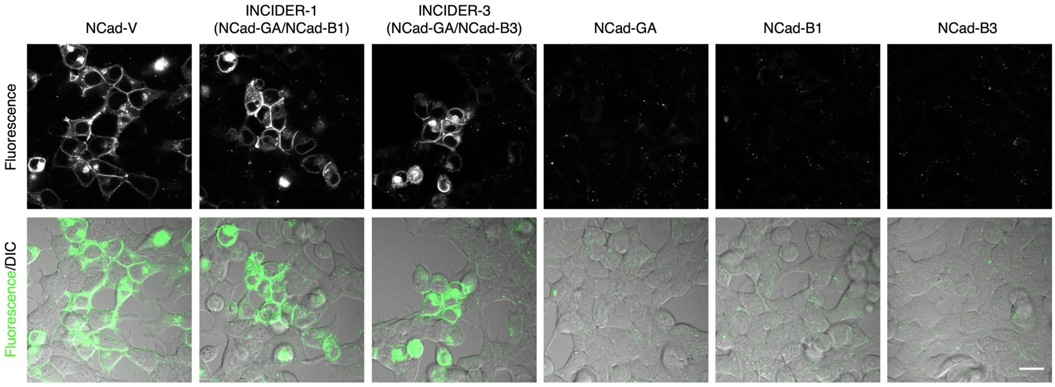

Supplementary Fig. 1 Co-expression of INCIDER components. Fluorescence images and merged images of fluorescence and differential interference contrast (DIC). HEK293T cells expressing the indicated constructs were observed using a confocal microscope. Co-expression of INCIDER components showed fluorescence. Scale bar, 20 μm.

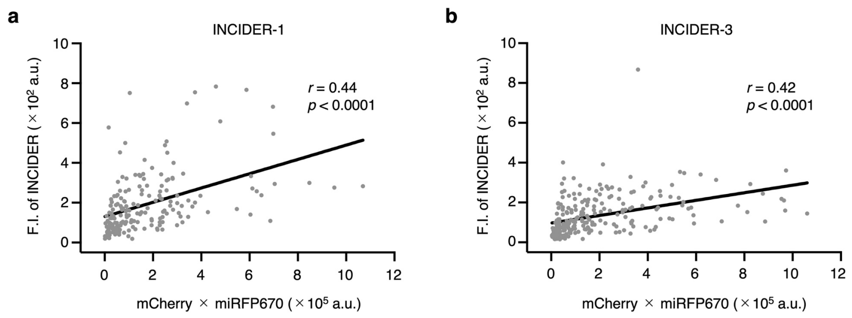

Supplementary Fig. 2 Correlation between INCIDER fluorescence intensity and the expression level. a, b Fluorescence intensity of INCIDER was plotted over the expression levels estimated by their expression marker FPs. Pearson's $r = 0.44$ for INCIDER-1 (**a**); $r = 0.42$ for INCIDER-3 (**b**). 189 (INCIDER-1) and 244 (INCIDER-3) cell adhesion sites were analyzed from two independent experiments.

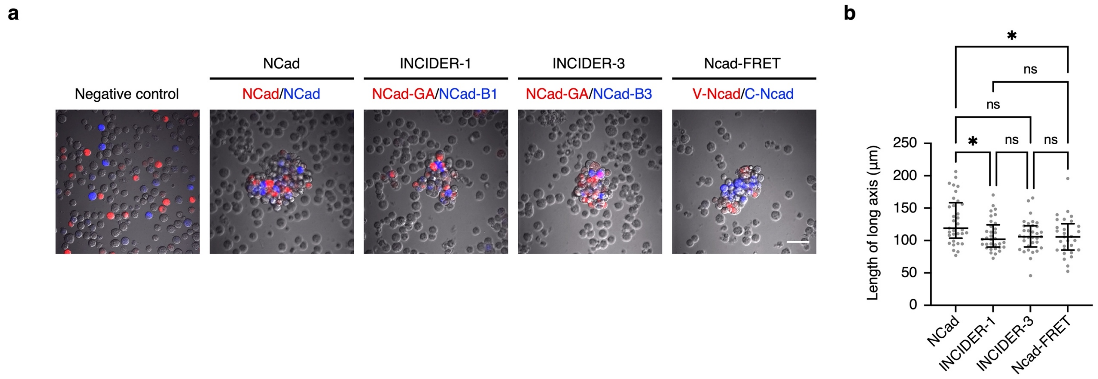

Supplementary Fig. 3 Effect of fluorescent protein insertion on the adhesive function. a K562 cells individually expressing the indicated constructs with mCherry-NLS or EBFP2-NLS were co-cultured and observed using a confocal microscope. Scale bar, 50 µm. **b** Lengths of long axis of formed cell aggregation were measured and compared. Results are presented as lower quartile (lower whisker), median (center line), and upper quartile (upper whisker). Significant differences were analyzed by Kruskal-Wallis test, followed by Dunn's multiple comparison test. *p < 0.05, ns indicates p > 0.05. 33 (NCad), 33 (INCIDER-1), 33 (INCIDER-3), and 29 (Ncad-FRET) cell aggregates from three independent experiments were analyzed.

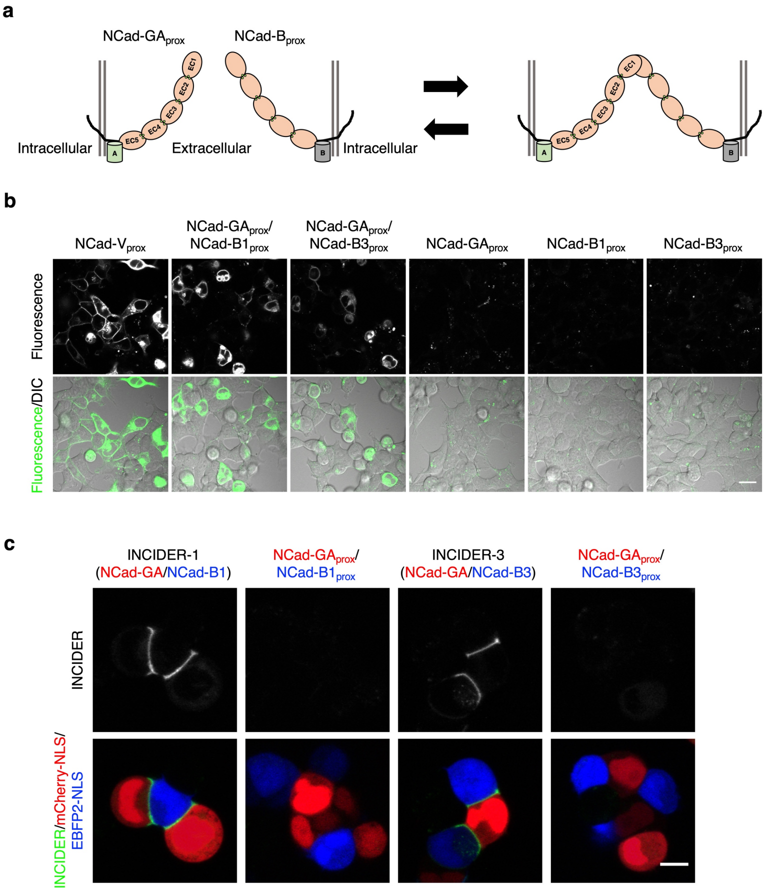

Supplementary Fig. 4 Importance of the ddGFP insertion site. a Schematics of ddGFP-inserted NCad, where the ddGFP is inserted far from the NCad interaction interface. In NCad-GA_{prox} and NCad-B_{prox} constructs, ddGFP-A and ddFP-B are inserted between an EC5 domain and a transmembrane region of NCad. **b** Co-expression of NCad-GA_{prox} and NCad-B_{prox} (NCad-$B1_{prox}$ or NCad-$B3_{prox}$) shows green fluorescence. HEK293T cells expressing the constructs indicated above were observed using a confocal microscope. Scale bar, 20 µm. **c** Intercellular NCad interaction is not visualized by NCad-GA_{prox}/NCad-B_{prox}. HEK293T cells individually expressing the indicated constructs were co-cultured and observed using a confocal microscope. NCad-GA or NCad-GA_{prox}-expressing cells and NCad-B or NCad-B_{prox}-expressing cells are marked by mCherry-NLS and EBFP2-NLS, respectively. Scale bar, 10 µm.

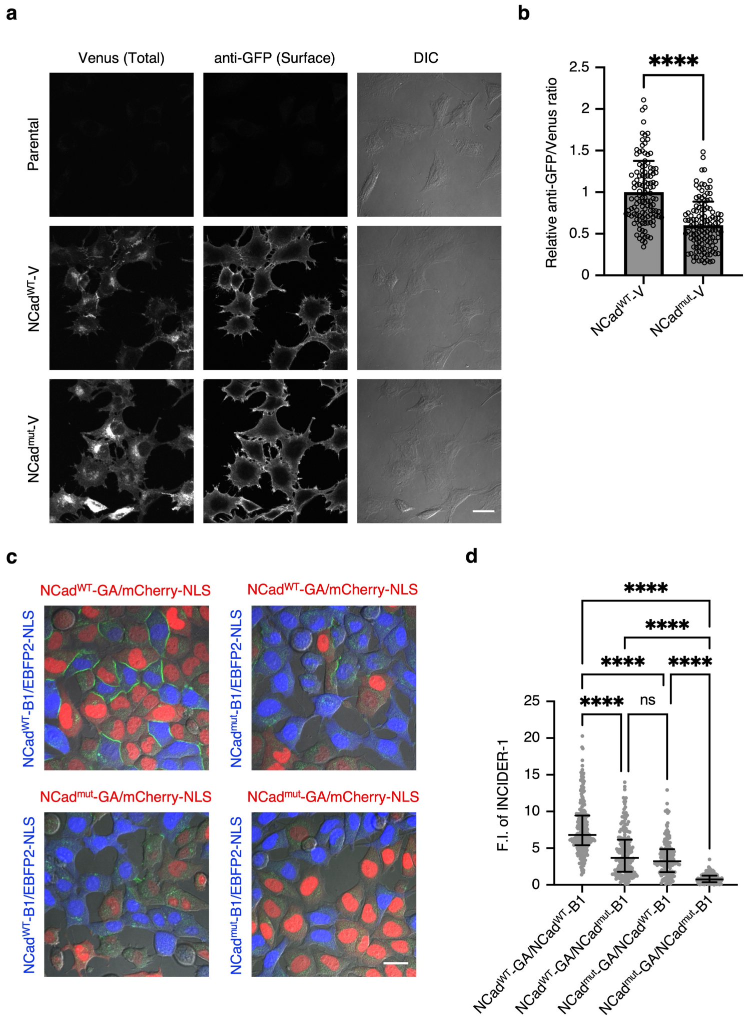

Supplementary Fig. 5 Characterization of L stable cell lines expressing wild-type and mutant NCad constructs. **a** Surface staining of L stable cell lines. The indicated cell lines were immunostained using an anti-GFP antibody without permeabilization. Scale bar, 20 µm. **b** Immunoreactive signals over Venus fluorescence signals were quantified and normalized by the mean value of NCad^{WT}-V. Data are presented as relative mean values ± SD. A significant difference was analyzed by Mann-Whitney *U*-test. ****$p < 0.0001$. 120 (NCad^{WT}-V) and 132 (NCad^{mut}-V) cells from three independent experiments were analyzed. **c** L stable cell lines were co-cultured as the indicated combination and observed using a confocal microscope. Scale bar, 20 µm. **d** INCIDER-1 fluorescence signals at cell–cell contact sites were quantified and compared. Data are presented as lower quartile (lower whisker), median (center line), and upper quartile (upper whisker). Significant differences were analyzed by Kruskal-Wallis test, followed by Dunn's multiple comparison test. ****$p < 0.0001$, ns indicates $p > 0.05$. 259 (NCad^{WT}-GA/NCad^{WT}-B1), 200 (NCad^{WT}-GA/NCad^{mut}-B1), 157 (NCad^{mut}-GA/NCad^{WT}-B1), and 195 (NCad^{mut}-GA/NCad^{mut}-B1) cell–cell contact sites from two independent experiments were analyzed.

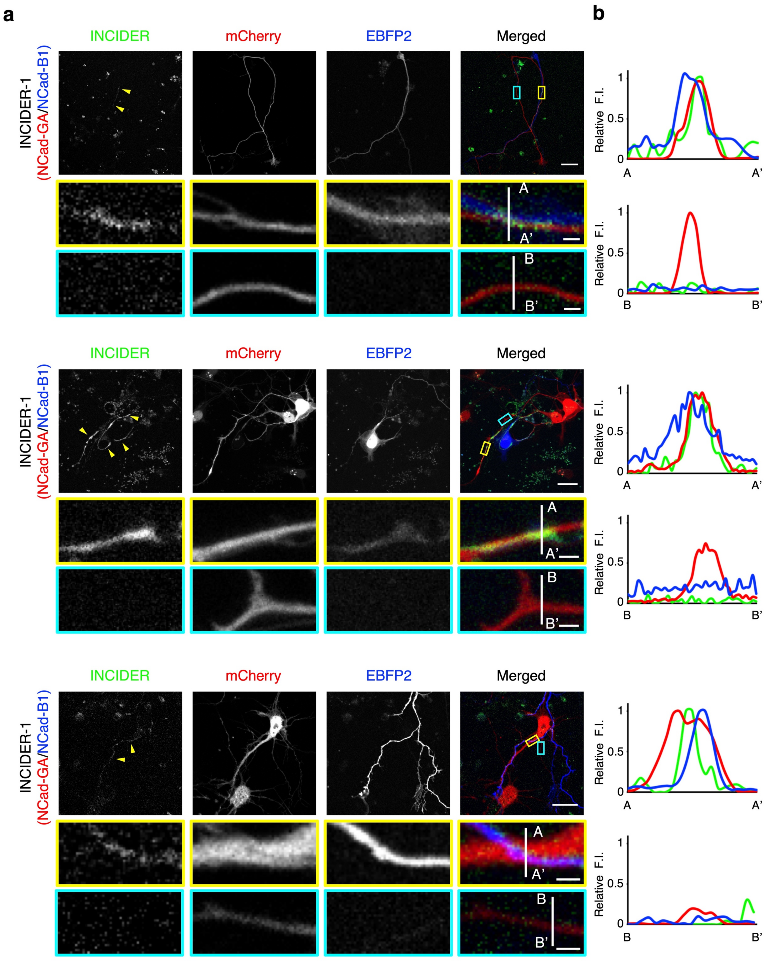

Supplementary Fig. 6 Examples of intercellular NCad interaction in neurons visualized by INCIDER-1. a Dissociated cortical neurons individually expressing INCIDER-1 components (NCad-GA and NCad-B1) were co-cultured and observed using a confocal microscope at 2 DIV. Scale bars, 20 µm (upper) and 2 µm (lower). **b** Fluorescence intensities along the white lines across a neuronal process of an NCad-GA-expressing neuron with (from A to A') or without (from B to B') a process of an NCad-B1-expressing neuron shown in **a** were measured. Relative fluorescence intensities of mCherry, EBFP2, and INCIDER are represented by red, blue, and green, respectively.

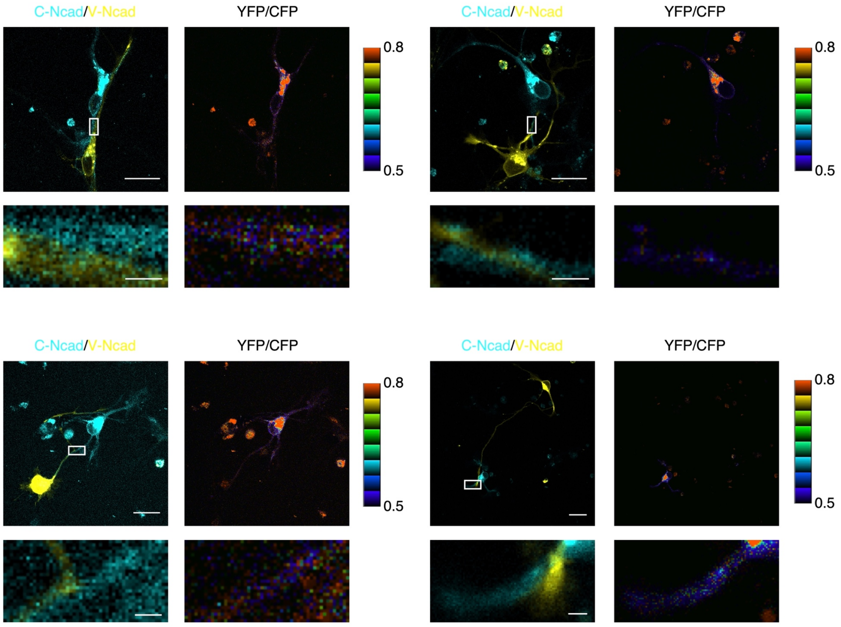

Supplementary Fig. 7 Application of Ncad-FRET to neurons. Dissociated cortical neurons individually expressing C-Ncad and V-Ncad were co-cultured and observed using a confocal microscope at 2 DIV. The FRET is displayed as a YFP/CFP ratio. Scale bars, 20 μm (upper) and 2 μm (lower). 18 fields of view from two independent experiments were observed.

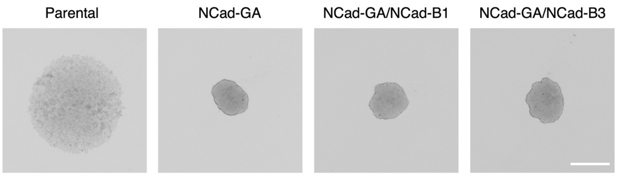

Supplementary Fig. 8 The adhesive function of INCIDERs. Parental L cells and L cells stably expressing the indicated NCad constructs were co-cultured in U-shaped dishes for 24 hours to form spheroids. Scale bar, 500 µm.

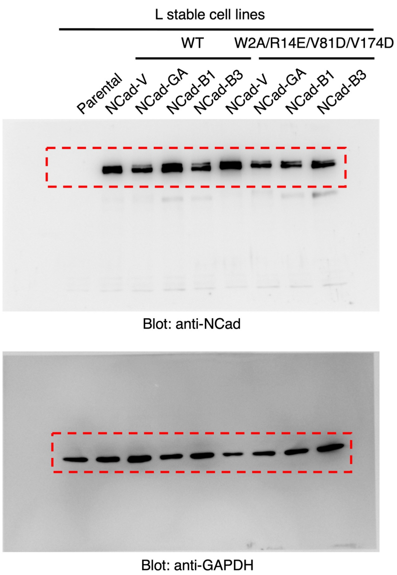

Supplementary Fig. 9 Uncropped images of western blot in Fig. 3a.
