# Supplementary material for: Development of intensiometric indicators for visualizing N-cadherin interaction across cells
Source: Commun Biol. 2022 Oct 7;5:1065. doi: 10.1038/s42003-022-04023-2 (PMC9546846; doi:10.1038/s42003-022-04023-2)
Supplement: Supplementary file 5 — Reporting Summary [file 42003_2022_4023_MOESM5_ESM.pdf]

nature portfolio

Corresponding author(s): Tomoki Matsuda

Last updated by author(s): Sep 16, 2022

Reporting Summary

Nature Portfolio wishes to improve the reproducibility of the work that we publish. This form provides structure for consistency and transparency in reporting. For further information on Nature Portfolio policies, see our Editorial Policies and the Editorial Policy Checklist.

Statistics

For all statistical analyses, confirm that the following items are present in the figure legend, table legend, main text, or Methods section.

n/a	Confirmed	
☐	☒	The exact sample size (*n*) for each experimental group/condition, given as a discrete number and unit of measurement
☐	☒	A statement on whether measurements were taken from distinct samples or whether the same sample was measured repeatedly
☐	☒	The statistical test(s) used AND whether they are one- or two-sided<br>*Only common tests should be described solely by name; describe more complex techniques in the Methods section.*
☒	☐	A description of all covariates tested
☒	☐	A description of any assumptions or corrections, such as tests of normality and adjustment for multiple comparisons
☐	☒	A full description of the statistical parameters including central tendency (e.g. means) or other basic estimates (e.g. regression coefficient) AND variation (e.g. standard deviation) or associated estimates of uncertainty (e.g. confidence intervals)
☐	☒	For null hypothesis testing, the test statistic (e.g. *F*, *t*, *r*) with confidence intervals, effect sizes, degrees of freedom and *P* value noted<br>*Give P values as exact values whenever suitable.*
☒	☐	For Bayesian analysis, information on the choice of priors and Markov chain Monte Carlo settings
☒	☐	For hierarchical and complex designs, identification of the appropriate level for tests and full reporting of outcomes
☒	☐	Estimates of effect sizes (e.g. Cohen's *d*, Pearson's *r*), indicating how they were calculated

Our web collection on statistics for biologists contains articles on many of the points above.

Software and code

Policy information about availability of computer code

Data collection	No software was used.
Data analysis	No software was used.

For manuscripts utilizing custom algorithms or software that are central to the research but not yet described in published literature, software must be made available to editors and reviewers. We strongly encourage code deposition in a community repository (e.g. GitHub). See the Nature Portfolio guidelines for submitting code & software for further information.

Data

Policy information about availability of data

All manuscripts must include a data availability statement. This statement should provide the following information, where applicable:

- Accession codes, unique identifiers, or web links for publicly available datasets
- A description of any restrictions on data availability
- For clinical datasets or third party data, please ensure that the statement adheres to our policy

The data and materials supporting this research are available from the authors on reasonable request.

Field-specific reporting

Please select the one below that is the best fit for your research. If you are not sure, read the appropriate sections before making your selection.

☒ Life sciences ☐ Behavioural & social sciences ☐ Ecological, evolutionary & environmental sciences

For a reference copy of the document with all sections, see nature.com/documents/nr-reporting-summary-flat.pdf

Life sciences study design

All studies must disclose on these points even when the disclosure is negative.

Sample size	Sample size calculations were not conducted. We provided the information of sample size of each experiment in each figure legends. We chose the sample sizes to support the conclusions we claimed in the manuscript.
Data exclusions	No data was excluded from the analyses.
Replication	All experiments performed on the manuscript were successfully replicated more than two times.
Randomization	Randomization was not relevant to the study.
Blinding	Blinding was not relevant to the study.

Reporting for specific materials, systems and methods

We require information from authors about some types of materials, experimental systems and methods used in many studies. Here, indicate whether each material, system or method listed is relevant to your study. If you are not sure if a list item applies to your research, read the appropriate section before selecting a response.

Materials & experimental systems

n/a	Involved in the study	
☐	☒	Antibodies
☐	☒	Eukaryotic cell lines
☒	☐	Palaeontology and archaeology
☐	☒	Animals and other organisms
☒	☐	Human research participants
☒	☐	Clinical data
☒	☐	Dual use research of concern

Methods

n/a	Involved in the study	
☒	☐	ChIP-seq
☒	☐	Flow cytometry
☒	☐	MRI-based neuroimaging

Antibodies

Antibodies used

anti-human N-cadherin antibody (Cat#M142, Takara Bio); anti-GAPDH antibody (Cat#sc32233, Santa Cruz Biotechnology); anti-GFP antibody (Cat#598, MBL)

Validation

<anti-human N-cadherin antibody>
• Rabbit polyclonal antibody against raised against the peptide (808-827) of human N-cadherin conjugated with KLH.
• This antibody cross reacts with mouse, bovine and chicken N-cadherin.
• This antibody does not react with human E-cadherin or P-cadherin.
• Immunohistochemical detection of N-cadherin on paraffin embedded or frozen tissue sections.
• Western blot analysis under non-reducing and non-heating condition.
<anti-GAPDH antibody>
• Mouse monoclonal antibody against raised against GAPDH purified from muscle of rabbit origin.
• This antibody cross reacts with mouse, rat, human, rabbit, Xenopus lavis origin.
• Western blot analysis.
• Immunoprecipitation.
• Immunofluorescence.
<anti-GFP antibody>
• Rabbit polyclonal antibody against raised against recombinant GFP.
• This antibody cross reacts with GFP variants (EBFP, SEBFP, ECFP, SECFP, EGFP, SEGFP, cpSEGFP, EYFP, Venus, cpVenus, R-pericom, and Sapphire).
• Western blot analysis.
• Immunoprecipitation.
• Immunocytochemistry.

Eukaryotic cell lines

Policy information about cell lines

Cell line source(s)	HEK293T cell line (RIKEN BRC), L cell line (ATCC), COS7 cell line (RIKEN BRC), and K562 cell line (RIKEN BRC).
Authentication	HEK293T, COS7, and K562 cell lines were authenticated by RIKEN.<br>L cell line was authenticated by ATCC.
Mycoplasma contamination	All cell lines were not tested for mycoplasma contamination.
Commonly misidentified lines<br>(See ICLAC register)	No commonly misidentified cell lines were used in this study.

Animals and other organisms

Policy information about studies involving animals; ARRIVE guidelines recommended for reporting animal research

Laboratory animals	Pregnant ICR mice (female)
Wild animals	This study did not involve wild animals.
Field-collected samples	This study did not involve samples collected from the field.
Ethics oversight	Animal experimentation was performed according to the Institutional Guidelines on Animal Experimentation at Keio University.

Note that full information on the approval of the study protocol must also be provided in the manuscript.
